# Fabrication of dual-functional composite yarns with a nanofibrous envelope using high throughput AC needleless and collectorless electrospinning

**DOI:** 10.1038/s41598-019-38557-z

**Published:** 2019-02-12

**Authors:** Jan Valtera, Tomas Kalous, Pavel Pokorny, Ondrej Batka, Martin Bilek, Jiri Chvojka, Petr Mikes, Eva Kuzelova Kostakova, Petr Zabka, Jana Ornstova, Jaroslav Beran, Andrei Stanishevsky, David Lukas

**Affiliations:** 1 Technical University of Liberec, Faculty of Mechanical Engineering, Liberec, 46117 Czech Republic; 2 Technical University of Liberec, Faculty of Textile Engineering, Liberec, 46117 Czech Republic; 3 Department of Physics, University of Alabama at Birmingham, 1300 University Boulevard, Birmingham, Alabama 35294 UK

## Abstract

Nanotechnologies allow the production of yarns containing nanofibres for use in composites, membranes and biomedical materials. Composite yarns with a conventional thread core for mechanical strength and a nanofibrous envelope for functionality, e.g. biological, catalytic, have many advantages. Until now, the production of such yarns has been technologically difficult. Here, we show an approach to composite yarn production whereby a plume of nanofibers generated by high throughput AC needleless and collectorless electrospinning is wound around a classic thread. In the resulting yarn, nanofibres can form up to 80% of its weight. Our yarn production speed was 10 m/min; testing showed this can be increased to 60 m/min. After the yarn was embedded into knitwear, scanning electron microscope images revealed an intact nanofibrous envelope of the composite yarn. Our results indicate that this production method could lead to the widespread production and use of composite nanofibrous yarns on an industrial scale.

## Introduction

The ability to form continuous yarns consisting of nanofibres is a significant breakthrough in electrospinning, as the nanofibre yarn can be woven or knitted into textiles. These textiles, in turn, can be used to create products with high added value, such as protective clothing, high performance fabrics, and composites, or employed in areas like tissue engineering^1^. Mastering the technology of nanofibre yarn production requires finding a way to transform polymeric jets into linear nanofibrous assemblies as well as attaining a satisfactory degree of productivity in the process. Most research and development to date has focused on DC electrospinning. The first attempt to produce a nanofibrous yarn in a laboratory using DC electrospinning was conducted using a rotary disc collector^2^. Theron *et al*. used a similar device to direct the nanofibres produced by a needle electrospinning process onto the periphery of a rotating disc-shaped collector^3^. The continuous production of nanoscale composite fibrils containing carbon nanotubes using electrospinning was described by Ko *et* *al*.^4^ Dalton *et al*.^5^ used two parallel and coaxial rotating rings as collectors to obtain short, twisted nanofibre yarns of 40 to 100 mm in length. Ali *et al*.^6^ developed a method for the continuous production of nanofibres using a rotating ring collector.

Another method for producing a composite yarn coated with nanofibres using DC needleless electrospinning was developed by Jirsak *et al*.^7^ and Yalcinkaya *et al*.^8^. A monofilament wound around the composite yarn ensured the resistance of the nanofibrous coat to abrasion. Whereas previously, 100% nanofibrous yarns were produced at the rate of tens of centimetres per minute, here the production rate of this composite yarn was two orders of magnitude higher. Recently, Viirsalu^9^ suggested a method by which nanofibres are aligned and twisted over the core yarn using a column with rotary air movement.

High throughput industrial sources of nanofibres based on DC electrospinning already exist at the industrial level: Elmarco (Czech Republic), FNM Fanavaran Nano-Meghyas (Iran), Inovenso (Turkey), SPUR (Czech Republic), Mecc (Japan), Nano azma (Iran) to mention a few. Highly productive needleless DC electrospinning methods, most of them based on free liquid surfaces and axially symmetric spinning electrodes, can be found in research works too^10–16^.

Beside DC electrospinning, its AC counterpart may be a better approach to the production of nanofibrous yarns on an industrial scale. Until the last decade, few studies were available on AC electrospinning and these were only focused on needle electrospinning processes driven by voltages ranging from 5 kV up to 10 kV only^17–19^. Our previous work has shown that AC electrospinning combined with an appropriately-shaped, needleless spinning-electrode is highly efficient at generating a dense plume of nanofibers at voltages above 30 kV^20^. Subsequent work has shown that the AC collectorless and needleless electrospinning method has the potential for many applications in biomedicine, textile industry, filtration, environmental sensors and enhanced catalists^21–24^. We have now created a high throughput method for producing composite yarns with a massive nanofibrous envelope.

## Results

### Description of an experimental production facility

**Figure 1:**
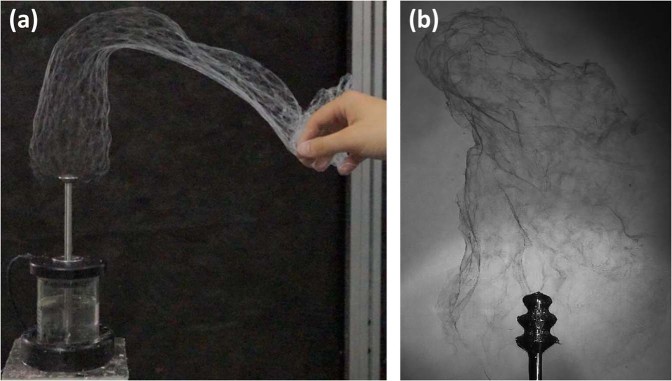
(**a**) The immediate product of AC electrospinning is a compact plume of nanofibres, which can be readily manipulated for further processing. The ability to grab and manipulate the plume by hand demonstrates that this method works without any electrically active collector. (**b**) The plume of nanofibres resembles fine smoke emerging from the AC electrospinning electrode. The spinning head of the electrode can be composed of three discs.

Our yarn production method is based on two inventions. First, the nanofibres are generated by a highly productive method of collectorless (Fig. 1a) as well as needleless AC electrospinning^20^. An important feature of AC spinning is that it creates a plume of nanofibres resembling a sleeve of interconnected nanofibres. These are transported by the electric wind in the direction of the axis of the rod-like spinning electrode (Fig. 1b) (Supplementary Information 1 and 2). This electrode, together with a screw pump and a polymer solution reservoir, form a single unit. The screw pump delivers the polymer solution to the top of the spinning electrode, where polymer jets are generated. The solution then flows down the outer surface of the electrode back into the polymer solution reservoir, ensuring a continuous process of AC electrospinning, with the top of the spinning electrode being steadily cleared of deposited fragments of solid nanofibres. Secondly, the plume of nanofibres^20^ produced by AC electrospinning is collected by the axially rotating and ballooning classic yarn^25–28^, which we call the *core yarn*. The axial rotation and ballooning is ensured by a set of two twirling devices. These devices have the capacity for virtually uninhibited axial rotation and ballooning of the yarn, even when a series of AC electrospinning units is used and the nanofibrous plumes cause a reduction in the motion and ballooning of the yarn.

**Figure 2:**
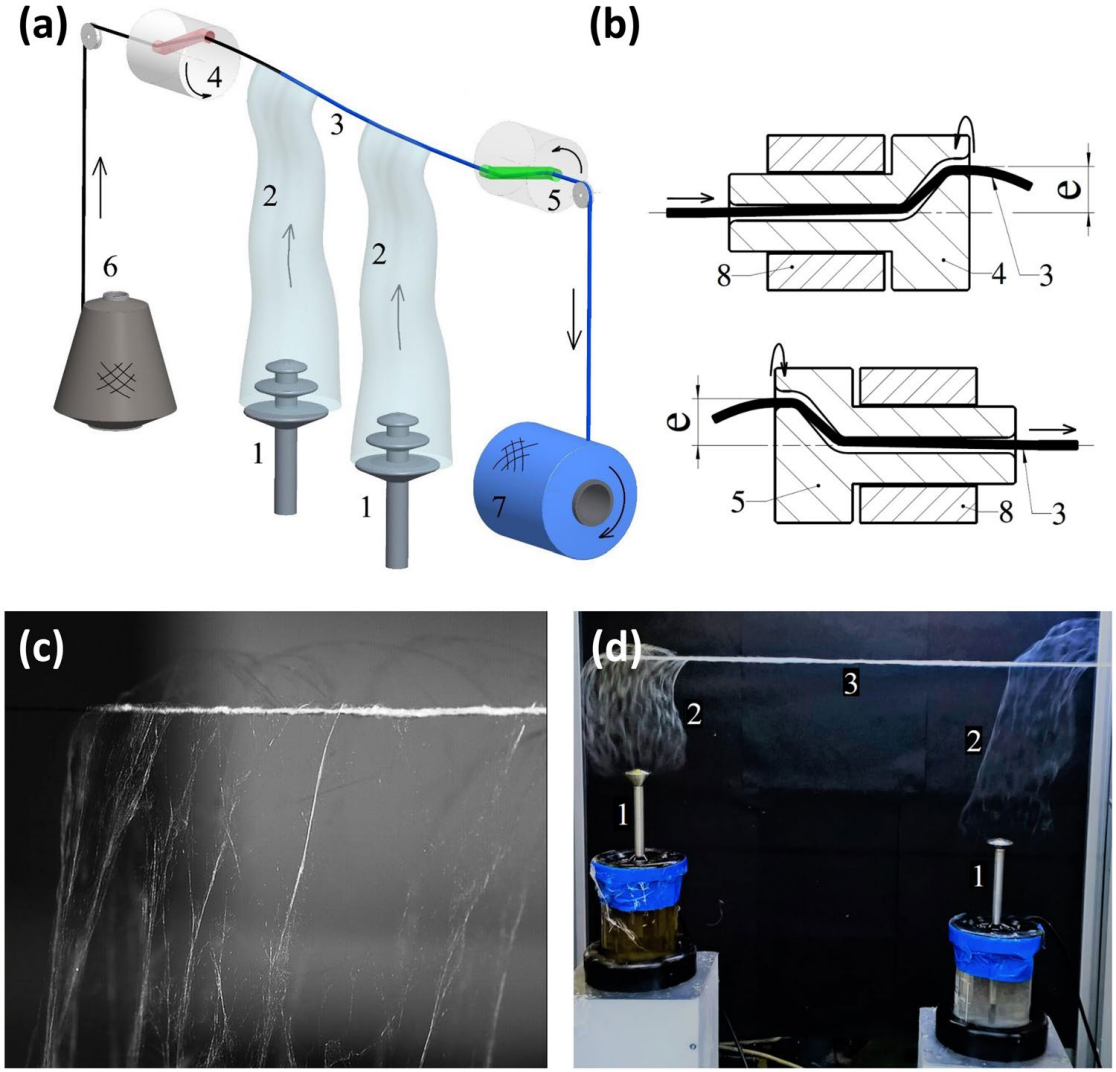
(**a**) A simplified schematic of the device: the AC spinning electrodes (1), the plumes of nanofibres (2), the core yarn being rewound from left to right (3), the first twirling device (4), the second twirling device (5), a storage coil with the core yarn coil (6), the output coil with the composite yarn (7). The yarn core is fed horizontally into the spinning space above the two vertically-oriented rod spinning-electrodes and is coated with nanofibres. (**b**) A simplified schematic of both twirling devices. The top image shows the first twirling device, which is comprised of a rotor (4) with a yarn chamber with an eccentric hole positioned with offset *e* from the axis of rotation, the static frame of the device (8), and the yarn (3) passing through the device. The bottom image shows a simplified schematic of the second twirling device (5). (**c**) The yarn core axis and the axis of the nanofibrous plume emitted from the spinning electrode are almost perpendicular to one another. (**d**) Two screw pumps integrated with the spinning electrode with disc like heads (1) and the polymer reservoir emitting nanofibrous plumes (2) which wrap around the ballooning yarn core (3).

The second twirling device (Fig. 2a,b) also allows for additional twisting of the nanofibrous envelope, wrapping it even more tightly around the core yarn. This creates a twist in the nanofibrous envelope, reduces yarn hairiness, and increases the adhesion between the core yarn and the nanofibrous envelope (see the discussion below in *The processability of composite yarns*). It also lowers the friction coefficient of the composite yarn’s surface during subsequent processing. This method of producing a massive nanofibrous envelope around the yarn core can be implemented with cores of a linear density ranging from tens up to thousands of dtex.

### Formation of the nanofibrous envelope around the core yarn

In our apparatus, the yarn core is fed horizontally into a spinning space at a distance of 150 mm above the spinning electrode, or set of spinning electrodes. The yarn core axis and the axis of the nanofibrous plume emitted from the spinning electrode are almost perpendicular to one another (Fig. 2c,d). The twirling devices are each composed of a rotating disc with an eccentric hole (see Fig. 2a,b for more details). The preloaded core of the yarn uncoils from the master bobbin, passes through the hole of the first disc, and then twists and loops into the shape of a balloon. Thanks to the local twisting and ballooning of the core yarn, the core is tightly enveloped by nanofibres at the area of contact of the core with the plume of nanofibres (Supplementary Information 3 and 4). Even when several plumes of nanofibres are wound up from a set of AC spinning units, the two co-rotating twirling devices ensure almost uninhibited rotation and ballooning of the yarn. The rotation and ballooning of the yarn depends on 7 factors: the mechanical properties, linear density, and diameter of the core yarn; the tension in the yarn; its winding speed; the angular speed of both twirling devices; and the eccentricity of their holes. The plume is formed by nanofibres which were originally electrically opposite. As a result of these attraction forces, a compact plume is formed^20^. This enables almost 100% of the nanofibre mass to be entrapped by the spinning and ballooning yarn core. Lastly, the resulting composite yarn is wound onto the output coil.

The process whereby the nanofibrous envelope is formed is complex. High-speed camera recordings show that the plume of nanofibres is not entirely plastic, but that it also exhibits a partially elastic behaviour that helps “tighten” the nanofibrous envelope around the core (Supplementary Information 5). This elasticity is a result of the structure of the plume, which consists of a network of mutually interconnected nanofibrous sections. Both in its elasticity, and also in structure, the plume resembles a fibrous web created by drum carders from staple fibres.

The ballooning, in general, arises from the circular motion of the ends of the core yarn around a solid axis, where at least one of the end points does not lie on the axis of rotation. Thanks to this, the core yarn entraps and wraps the plume of nanofibres around itself. The ballooning yarn creates an imaginary rotating surface called a yarn balloon (Supplementary Information 6–8). The effect of the tensile force in the yarn on the yarn maximum balloon radius was studied using the Migushov model^25–28^. The model takes into account the effect of centrifugal forces, air drag, and the Coriolis force, and assumes that the yarn is ideally flexible, uniform and non-stretchable. The angular speed of the rotating yarn and yarn winding speed are modelled as constant.

**Figure 3:**
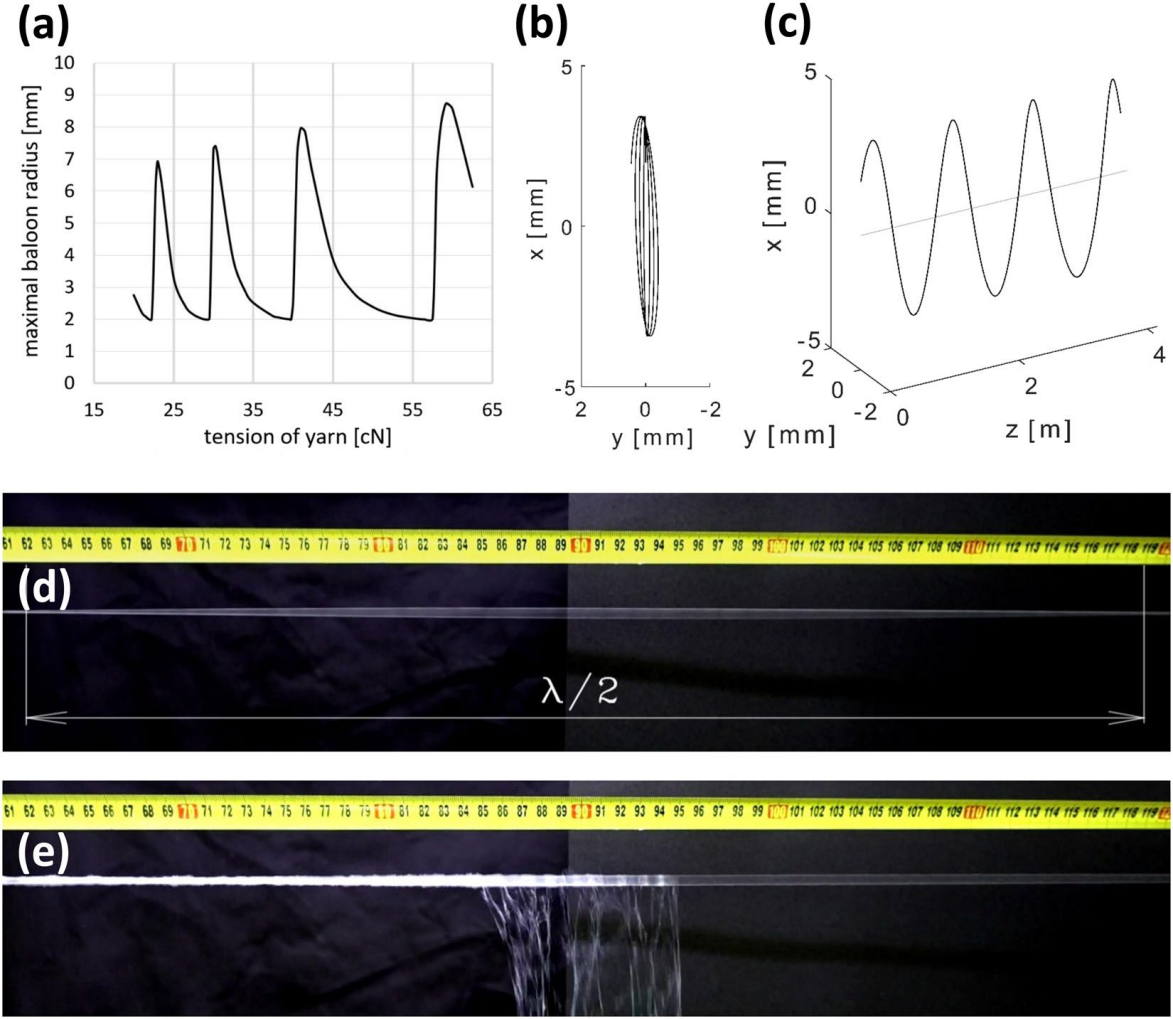
(**a**) The maximum radius of the balloon depending on the tension of the yarn. (**b**) The shape of the ballooning yarn in the axial view. (**c**) The shape of the ballooning yarn between two twirling devices 4.5 m apart, under a constant yarn stress of 33 cN. (**d**) A section of the ballooning yarn inside the spinning space. The distance between two nodes in the balloon is 0.57 m. (**e**) When the ballooning yarn is weighed down with the plume of nanofibres, the balloon nodes cease to be clearly visible.

Figure 3a shows the dependence of the maximum radius of the balloon on the tension of the yarn, given the following parameter values: a rotational speed of 5000 RPM for both twirling devices; a yarn winding speed of 10 m/min; a core yarn fineness of 330 dtex; a 2 mm eccentricity of the guiding eyelet in the twirling device; a distance of 4.5 m between the twirling devices; and a yarn stress of 33 cN. The model shows that increasing the yarn tension decreases the balloon radius until the radius reaches a minimal value (2 mm in our model), at which point it abruptly jumps to the next maximum value, each maximum being slightly higher than the previous one. This phenomenon repeats as the yarn tension is increased. Migushov’s model predicts the spatial layout of the ballooning yarn (Fig. 3b,c). The model predicts a distance of 0.63 m between adjacent nodal points of the yarn balloon, which corresponds with our experimental measurements of 0.57 ± 0.05 m (Fig. 3d,e).

### SEM investigation

**Figure 4:**
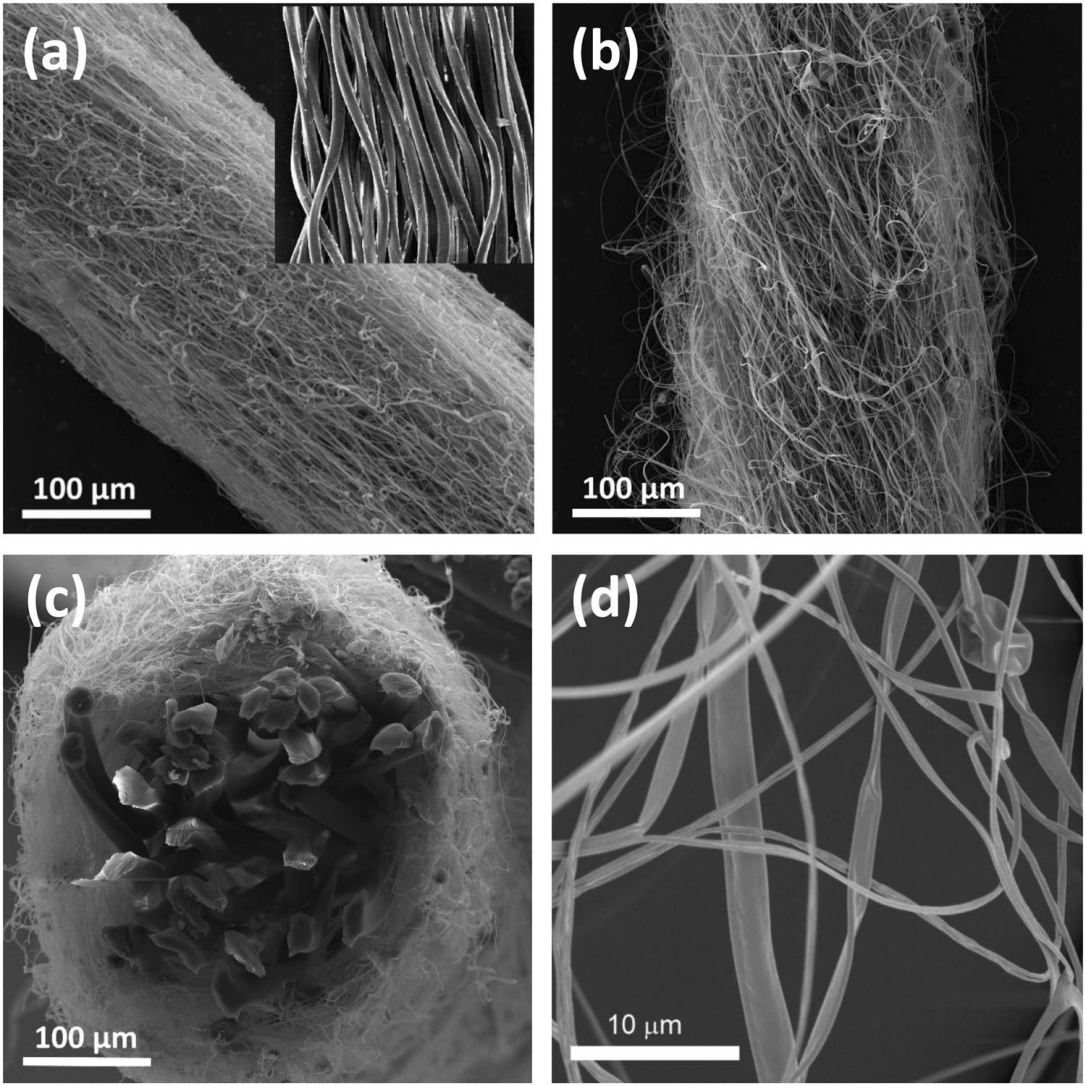
(**a**) A composite yarn with almost zero twist and a bare core yarn. (**b**) The nanofibre envelope with a twist value of about 10^3^ m^−1^. (**c**) A cross section of a composite yarn with a polyester (PES) multifilament core with a linear weight of 330 dtex, and a nanofibrous envelope made with polyamide 6 (PA6). (**d**) A detailed view of the structure of a nanofibre envelope showing belts, beads and inter-twined fibers. Fibres are tortuous (inter-twined) due to the mechanism of their creation, in which positively and negatively charged jet segments are mutually attracted.

Scanning electron microscope (SEM) images (Fig. 4) show composite yarns where the nanofibre envelopes were each given a different degree of additional twisting, resulting in a different numbers of twists per unit of length (twist value). The twist value depends on the angular frequency of the twirling devices. The composite yarn without any additional twist is depicted in Fig. 4a, while Fig. 4b shows a composite yarn with a twist value of approximately 10^3^ m^−1^. Figure 4c shows the cross-section of the composite nanofibrous yarn, and the morphology with belts, beads and doubled fibres of the nanofibrous envelope is shown in detail in Fig. 4d.

### The processability of composite yarns

The processability of composite yarns into woven or knitted fabrics was evaluated using three tests of their mechanical resistance. First, we measured the friction coefficient of the nanofibrous envelope. Second, we evaluated the adhesion of this envelope to the core yarn. Lastly, we knitted a composite yarn sample into a textile fabric. The parameters of the six samples of composite yarns we tested are listed in Table 1.

**Table 1:** The material parameters of the six tested samples of composite yarns.

Sample No.	Material of the core yarn	Material of the envelope	Rotational speed of the second twirling device (RPM)
1	Polyester (PES) 263 dtex	Poly(vinyl butyral) (PVB)	0
2	Polyester (PES) 263 dtex	Poly(vinyl butyral) (PVB)	5 000
3	Polyester (PES) 263 dtex	Poly(vinyl butyral) (PVB)	7 700
4	Polyamide 6,6 (PA 6,6) 122 dtex	Polyamide 6 (PA6)	6 000
5	Polyamide 6,6 (PA 6,6) 36 dtex	Polyamide 6 (PA6)	10 000
6	Polyamide 6,6 (PA 6,6) 22 dtex	Polyamide 6 (PA6)	15 000

**Figure 5:**
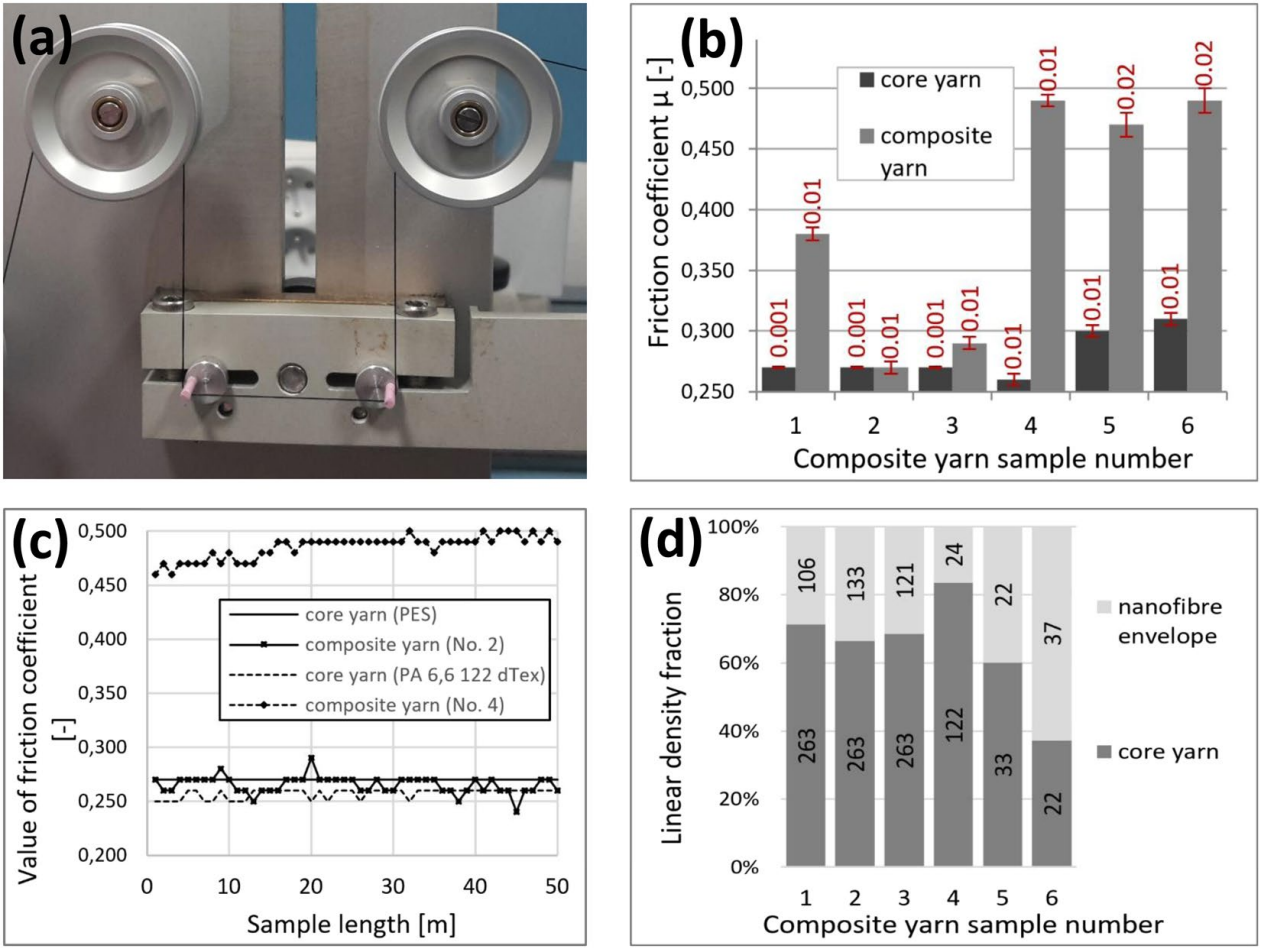
(**a**) A Constant Tension Tester. The composite yarn is wrapped around two ceramic rods with a total wrapping angle of 180°. (**b**) A comparison of the friction coefficients of the six composite yarn samples (grey bars) and six samples of their bare yarn cores (black bars), with standard deviations. For each uncoated and coated sample 50 measurements were carried out. Samples No. 1–3 have a polyester (PES) core and a polyvinylbutyral (PVB) envelope. Samples No. 4–6 have a polyamide 6,6 (PA 6,6) core and a polyamide 6 (PA6) envelope. **(c)** The typical friction coefficient values of a series of successive sections of a composite yarn for samples No. 2 and No. 4. For each sample 50 measurements were carried out. All samples were 50 m long. (**d**) The linear density fractions of the core and envelope fibres in composite yarn samples. The linear density values of the core and envelope fibres are introduced inside the bars in dtex.

First, we established the friction coefficients of the six composite yarn samples and six core yarn samples. The tension of the yarn sample was measured as it was being wound around a static element with a defined wrapping angle (Fig. 5a). We measured 50 yarns, each sample 1 m long, and then determined the coefficient of friction *µ* using the Capstan friction equation^29^. The results, including standard deviations, are shown in Fig. 5b. Sample No. 1 has higher friction coefficient due to its bristly nanofibrous envelope since the second twirling device was set to 0 RPM (Table 1). The bare polyamide 6,6 (PA 6,6) core yarns exhibited increasing friction coefficient values with their decreasing linear density (Table 1, Fig. 5b). For comparison, the typical friction coefficient values of a series of successive sections of a composite yarn are shown in Fig. 5c. Our results indicated that with the appropriate value of additional twisting, ensured by the second twirling device, the morphology of the nanofibre envelope is changed. This is evidenced by a decrease in the friction coefficient for polyvinylbutyral (PVB) envelopes, i.e. samples No. 2 and 3. For example, sample No. 2 had a friction coefficient value of *μ* = 0.27. On the other hand, for samples with a polyamide 6 (PA6) envelope, i.e. samples No. 4–6, there is a significant increase in the friction coefficient values up to 0.47 and 0.49, compared to their yarn core, independent of additional twisting. This increase is approximately 48% in comparison to the PVB coated composite yarns, and is probably related to the polymer type and core yarn fineness. Polymer-polymer friction has been studied on a level of interactions between individual chains by Maeda *et al*.^30^. They revealed that friction coefficients depend by a complex manner on dynamics of the outermost polymer chains at sharing interfaces. Typical friction coefficient values for various fibres and yarns passing over hard steel, porcelain, fibre pulley and ceramic guides were introduced by Morton *et al*.^31^. In addition, Fig. 5d shows the fineness of the individual composite yarn samples in dtex, as well as the linear density fraction in composite yarns No. 1–6. For instance, the overall fineness of sample No. 6 is 59 dtex. The yarn core (22 dtex) forms only 37% of the total weight, and the remaining 63% is composed of nanofibres (37 dtex).

**Figure 6:**
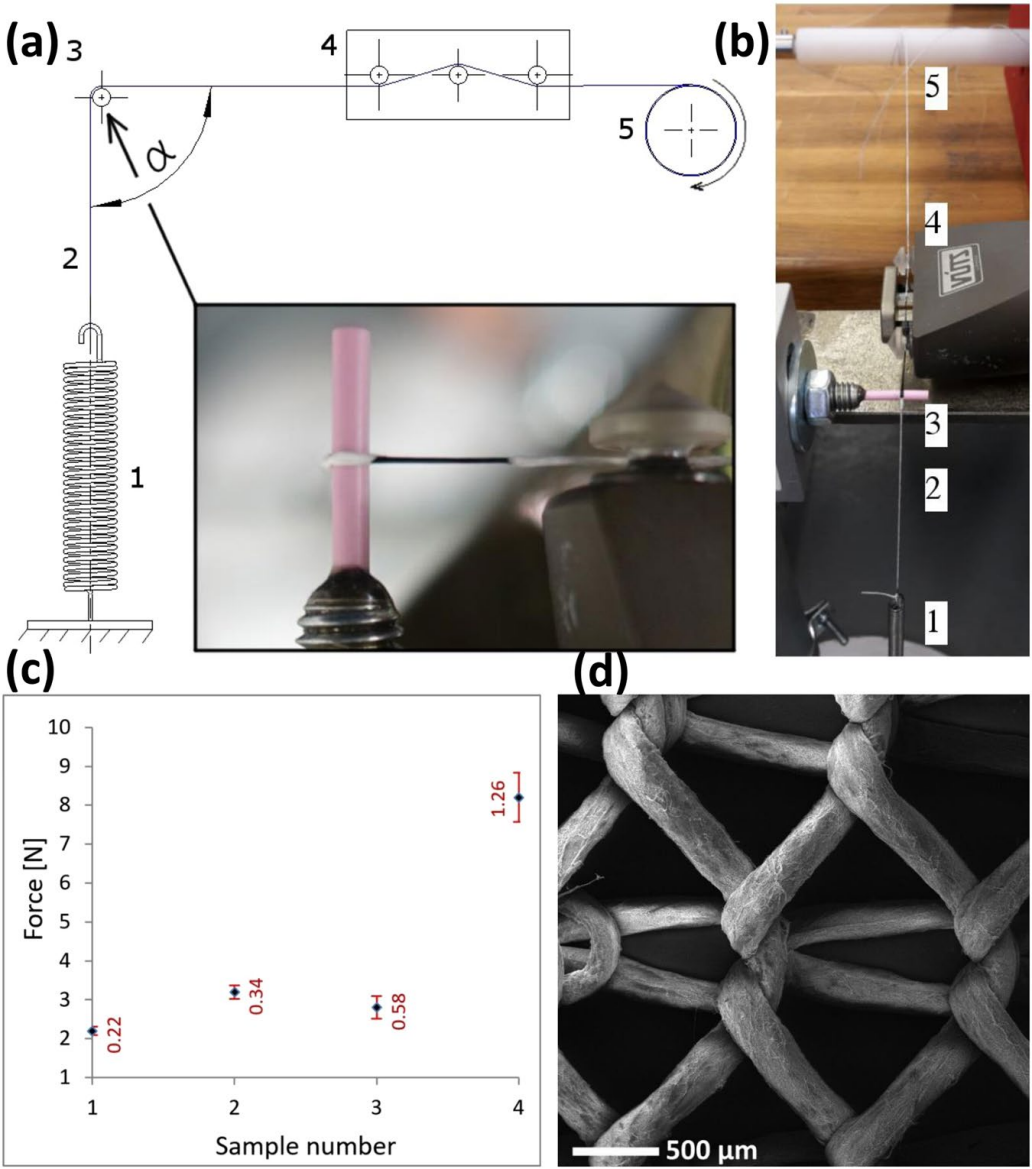
(**a**) A diagram of the device measuring the envelope-core adhesion. (Inset) A photograph of the composite yarn (sample No. 1) at the moment when the envelope was damaged: (1) a spring, (2) the yarn to be tested, (3) a ceramic wire, (4) a tension sensor, (5) the shaft of the winding device. (**b**) The measuring device at the moment when the nanofibre envelope was detached from the yarn core (sample No. 1). (**c**) The force values and standard deviation bars at which the nanofibre envelope was detached from the core yarn. Ten measurements were carried out for each of the samples No. 1–6. (**d**) A SEM microphotograph of knitwear made with a composite nanofibrous yarn indentical to sample No. 4.

Second, we tested the adhesion of the nanofibre envelope to the yarn core (i.e., envelope-core adhesion). Adhesion is understood here in its general meaning as the tendency of dissimilar materials to cling to one another, which is caused by van der Waals forces between fibres surfaces and mechanical effects due to fibres becoming entangled. This test was done using a custom measuring device equipped with a commercially available and calibrated yarn tension sensor (Fig. 6a,b). When testing samples No. 5 and 6, the core yarn failed, together with the envelope, when the tension in the composite yarn exceeded 10 N. This prevented us from measuring the envelope-core adhesion for these samples. Thus samples No. 5 and 6 are not listed in our results (Fig. 6c). The envelope-core adhesion is lowest in the the composite yarn with a PVB envelope without any additional twist (sample No. 1). Here, the nanofibre envelope broke at yarn tension value of 2.2 N. Samples No. 2 and 3, which were given some additional twist by the second twirling device, demonstrated a higher envelope-core adhesion within values of 2.8 N and 3.2 N. In our measurements the Polyamide 6 envelope (sample No. 4) had an envelope-core adhesion approximately 3 times higher than the envelopes of samples No. 1–3 made with PVB (Fig. 6c).

Lastly, a knitting process tested the processability of composite yarns into textile fabrics. For this workability test, a yarn identical to sample No. 4 was used. The result is shown in Fig. 6d. The PA6 envelope suffered no mechanical damage when the loops of the knitted fabrics were tightened.

## Discussion

The AC electrospinning variant introduced here is a high throughput method to produce nanofibrous material. This method works without any electrically active collector and therefore the plume of nanofibres can easily be coupled to another technological unit. In addition, the immediate product of AC electrospinning is a compact plume of nanofibres, which can be readily manipulated for further processing. As an example of this, we have shown a promising application of the nanofibrous plume, namely the production of composite nanofibrous yarns.

Our apparatus has great potential because it enables the linear density of the yarn core to be reduced to as little as 10 dtex, if required. Subsequent testing suggested that in such a case the nanofibre envelope may form more than 80% of the yarn weight. Our apparatus allows for more spinning units to be added, enabling an increase in the production rate of the composite yarn, provided the yarn core is given a large enough nanofibre coat. The production speed of composite yarns in the spinning apparatus described here was 10 m/min. Further tests showed that with a set of three spinning electrodes, a homogeneous wrapping of the yarn core can be created, even at a production speed of 60 m/min. A suitable combination of spinning units containing different types of polymer solutions also enables the creation of a multilayered composite nanofibrous yarn with several special properties such as, biological, catalytic or sorption. Given that our approach to electrospinning does not require an electrically active collector, while, at the same time, there is both scalability of our electrospinning apparatus, as well as high throughput, we anticipate that this approach could lead to the widespread production and use of composite nanofibrous yarns on an industrial scale.

## Methods

### AC Electrospinning apparatus

The AC needleless electrospinning setup consisted of a KGUG 36 high-voltage transformer with a convertion ratio of 36000 V/230 V, ABB-Asea Brown Boveri (Switzerland). The output voltage was controlled by the ESS 104 variable autotransformer, Thalheimer Transformatorenwerke Gmbh, (Germany), designed for a 230 V AC input and an output of 0–250 V. The maximum output current was 4 A with a capacity of 1.2 KVA. This AC power supply operated at 50 Hz at an effective voltage of up to a maximum of 32 kV, at which all the experiments were performed. The rod spinning-electrode was integrated into a polymer solution hopper with a screw pump, Technical University of Liberec (Czech Republic). The length of the spinning electrode was 100 mm and the diameter of the spinning head was 15 or 20 mm, for polyamide 6 or polyvinylbutyral solutions respectively. The polymer solution was fed to the top of the spinning electrode through a 3 mm coaxial channel. The speeds of the two twirling devices, Technical University of Liberec (Czech Republic) ranged from 0–20,000 RPM, depending on the type of core yarn and the polymer solution used (see Table 1 for details). When a single spinning unit was used, the production rate of the polyvinylbutyral (PVB) envelope was 20 m/min, while the polyamide 6 (PA6) envelope was produced at the speed of 10 m/min. After being coated with nanofibres, the newly-formed composite yarn passed through a 2.2 m long drying zone. The zone consisted of two heated tubes specially designed for this application by the manufacturer ELKOP Technik s.r.o (Czech Republic). The temperature in both tubes could be adjusted independently of each other. The overall power of one heating tube was 1000 W and the dimensions were: inner diameter 30 mm, outer diameter 55 mm and length 1100 mm. In addition, a pre-heated air flow into the heating system was ensured by the hot air-blower Mistral 6, Leister Technologies AG (Switzerland). The air temperature in the drying zone was maintained in the range of 85–110 °C. The temperature was lower (85–90 °C) for the PA6 envelope, and higher (100–110 °C) for the PVB nanofibrous envelope. The total length of the drying zone was 2,000 mm. The yarn then passed through a second twirling device, which smoothened the nanofibre envelope on the yarn core. The composite yarn then travelled to an output coil where it was wound up in a guided manner. The movement of the plume of nanofibres was detected using a high-speed camera system i-SPEED 3 with an F-mount lens connection and a recording frequency of 2000 Hz to obtain a maximum picture resolution of 1280 × 1024 px. The light source used was an ILP-1 with a discharge lamp of 120 W at a temperature of 5 600 K and was focused using an optical cable. An analysis of the video recording was carried out using I-SPEED Suite software.

### Materials

The polyvinylbutyral (PVB), Mowital® B 60 H, with an average molecular weight of 60,000 amu, was obtained from Kuraray America, Inc. A 12 wt% solution of PVB was prepared in ethanol from Tereos TTD and n-propanol from PENTA (Czech Republic) (4:1 v/v). The polyamide 6 (PA6) Ultramid B 27 was supplied by BASF. A 10 wt% solution of PA6 was prepared in a 9:1 v/v blend of acetic and formic acid from PENTA (Czech Republic). Both polymeric solutions were stirred with a magnetic stirrer (250 RPM) at room temperature (24 °C) for 20 hours. The following multifilament core yarns were used: polyester (PES) 330 dtex, ELASTEX Krnov (Czech Republic), PES 263 dtex, SETILA s.a. (France), polyamide 6,6 (PA 6,6) 122 dtex and PA 6,6 36 dtex by PPH LEGS SP. Z o.o. (Poland), PA 6,6 22 dtex by AQUAFIL s.p.a., Arco (Italy).

### SEM investigation

Figure 4d was taken with the UHR-FE-SEM Zeiss Ultra Plus microscope (Germany) using integrated In-Lens secondary electron detector at an accelerating voltage of 2 kV; an aperture 20 um; a working distance of 4.6 mm; a pixel size 44.66 nm. The sample was not modified to make it conductive. All other SEM images were taken with the VEGA3 SBU – EasyProbe microscope, Tescan (Czech Republic) equipped with a Tungsten heated cathode as the electron gun and the secondary electron detector Everhart-Thornley type (YAG Crystal). The images were takenat High Vacuum Mode (SE) at a resolution of 8 nm at 3 kV.

### Friction coefficient measurements

The friction coefficient *μ* was measured using a Constant Tension Tester (CTT) from Lawson-Hemphill, Inc. (USA). To obtain the friction coefficients shown in Fig. 5b,c we measured 50 yarns, each 50 m in length, for each of the coated and uncoated samples No. 1–6. Composite yarns with a polyvinylbutyral (PVB) nanofibrous envelope were created with one spinning electrode while a polyamide 6 (PA6) envelope was created using two spinning electrodes. Measurements were carried out at 21.1 °C and at a humidity of 64% RH. The yarn was wound around two static ceramic rods Ascotex Ltd (United Kingdom) with a 3 mm diameter and a total wrap angle of 180°, see Fig. 5a. The distance betweem the rods was 100 mm. When rewinding the yarn on the CTT, the yarn was preloaded, depending on its fineness, with a standard value of 1 g/tex, and rewound at a constant speed of 100 m/min. The measurements were averaged and the standard deviation was calculated.

### Adhesion measurement

For each composite yarn (samples No. 1–6), ten measurements were carried out. One end of the 1500 mm long yarn piece to be tested was fastened to a tension spring eyelet, and the other end to a winding device shaft. In between the eylet and the shaft lay a ceramic guiding element, Ascotex Ltd. (United Kingdom), with a diameter of 3 mm. The yarn was wound at an angle α = 90° around this ceramic guiding element, and then passed through a calibrated yarn tension sensor, VUTS a.s. (Czech Republic), with a measuring range of 0–1000 cN. During the test, the yarn was wound around the winding device shaft at a constant velocity *v*_1_ = 10 m/min and the elongation of the spring increased the tensile force in the yarn until the nanofibre envelope broke at the place where the composite yarn was wound over the ceramic guiding element (Fig. 6a,b). At the moment of the first failure of the nanofibre envelope, the value of the tensile force was read off the yarn tension sensor.

### Knitting machine

The yarn processability was tested on a Shima Seiki NewSES 122 FF knitting machine (Japan) with a gauge 18E, i.e., 18 needles per English inch. Testing of the processability took place at a knitting rate of 0.35 metres of a line per second. The yarn was fed to the machine using a cone bobbin to make unwinding during the knitting process easier. The knitting process was facilitated by a feeding mechanism. First, a starting-up course and a draw course were created from a conventionally produced yarn. Only then did we introduce the composite nanofibrous yarn. A single-jersey knit fabric of the textile wave twill was created.

## Supplementary information

Supplementary information 1

Supplementary information 2

Supplementary information 3

Supplementary information 4

Supplementary information 5

Supplementary information 6

Supplementary information 7

Supplementary information 8

Overview of supplementary information
