# Supplementary material for: Fabrication of dual-functional composite yarns with a nanofibrous envelope using high throughput AC needleless and collectorless electrospinning
Source: Sci Rep. 2019 Feb 12;9:1801. doi: 10.1038/s41598-019-38557-z (PMC6372629; doi:10.1038/s41598-019-38557-z)
Supplement: Supplementary file 9 — Overview of supplementary information [file 41598_2019_38557_MOESM9_ESM.pdf]

Fabrication of dual-functional composite yarns with a nanofibrous envelope using high throughput AC needleless and collectorless electrospinning

Jan Valtera[1], Tomas Kalous[2], Pavel Pokorny[2], Ondrej Batka[1], Martin Bilek[1], Jiri Chvojka[2], Petr Mikes[2], Eva Kuzelova Kostakova[2], Petr Zabka[1], Jana Ornstova[2], Jaroslav Beran[1], Andrei Stanishevsky[3] and David Lukas[2,*]

[1]Technical University of Liberec, Faculty of Mechanical Engineering, Liberec, 46117, Czech Republic

[2]Technical University of Liberec, Faculty of Textile Engineering, Liberec, 46117, Czech Republic

[3]Department of Physics, University of Alabama at Birmingham, 1300 University Boulevard, Birmingham, Alabama 35294

Supplementary Information:

Supplementary information 1 – The nanofibre plume produced by the AC spinning method.

Supplementary information 2 – The nanofibre plume produced by the AC spinning method; high-speed camera recording.

Supplementary information 3 – The nanofibre plume wrapping around the ballooning yarn core.

Supplementary information 4 – The nanofibre plume wrapping around the ballooning yarn core; high-speed camera recording.

Supplementary information 5 – The process of the nanofibrous plume wrapping around the ballooning yarn core, documenting its partial elasticity; video recording from a high-speed camera.

Supplementary information 6 – Ballooning core of the yarn when the yarn stress is changing.

Supplementary information 7 – The ballooning yarn core; high-speed camera recording.

Supplementary information 8 – The ballooning yarn core being enwrapped by a plume of nanofibres when the yarn stress is changing.
